# Analysis of hygroscopic self-shaping wood at large scale for curved mass timber structures

**DOI:** 10.1126/sciadv.aax1311

**Published:** 2019-09-13

**Authors:** Philippe Grönquist, Dylan Wood, Mohammad M. Hassani, Falk K. Wittel, Achim Menges, Markus Rüggeberg

**Affiliations:** 1 Laboratory for Cellulose & Wood Materials, Empa, Überlandstrasse 129, 8600 Dübendorf, Switzerland.; 2 Institute for Building Materials, ETH Zurich, Stefano-Franscini-Platz 3, 8093 Zürich, Switzerland.; 3 Institute for Computational Design and Construction, University of Stuttgart, Keplerstrasse 11, 70174 Stuttgart, Germany.

## Abstract

The growing timber manufacturing industry faces challenges due to increasing geometric complexity of architectural designs. Complex and structurally efficient curved geometries are nowadays easily designed but still involve intensive manufacturing and excessive machining. We propose an efficient form-giving mechanism for large-scale curved mass timber by using bilayered wood structures capable of self-shaping by moisture content changes. The challenge lies in the requirement of profound material knowledge for analysis and prediction of the deformation in function of setup and boundary conditions. Using time- and moisture-dependent mechanical simulations, we demonstrate the contributions of different wood-specific deformation mechanisms on the self-shaping of large-scale elements. Our results outline how to address problems such as shape prediction, sharp moisture gradients, and natural variability in material parameters in light of an efficient industrial manufacturing.

## Teaser

Insight into mechanics of biomimetic self-shaping wood leads to revolutionizing manufacturing of curved structural timber.

## INTRODUCTION

Wood is a vastly abundant, sustainable, and well-performing construction material. From its biological origin, it inherits material characteristics such as anisotropy and hygroscopy, which are still seen as drawbacks in wood technology, as they limit traditional uses. In nature, many biological systems have been identified as being capable of large shape changes in response to changes in humidity. The unique combination of anisotropy and hygroscopy with their smart structure makes this possible (*1*–*13*). Large shape changes can reciprocally be achieved by wood cross-ply laminates with layup (0°/90°), referred to as bilayers, when changing moisture content by either drying or wetting. Making use of the seeming inherent disadvantage of swelling and shrinkage of wood, thin wooden bilayers with fast dynamic responsiveness have recently been highlighted for diverse applications, predominantly as functional elements in biomimetic architecture (*14*–*23*).

**Fig. 1:**
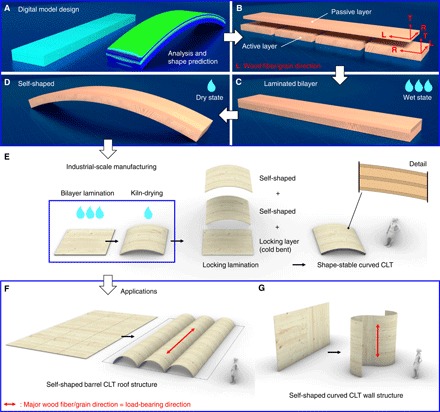
Self-shaping wood bilayer application at large scale. (**A** to **D**) Analysis and design process at laboratory scale. (**E** to **G**) Industrial scale, same thickness but increased length and width of bilayers toward plate geometries. (A) Parametric digital model and FE analysis for shape prediction of arbitrary configuration. (B) Example bilayer strip configuration (here, European beech wood) with passive and active layer components with wood anatomical directions radial (R), tangential (T), and longitudinal (L). (C) Laminated wooden bilayer strip in initial, wet, and flat shape. (D) Curved self-shaped bilayer after drying. (E) Industrial-scale manufacturing (here, Norway spruce wood) in plate and shell configurations. Plates can be air-dried or kiln-dried to achieve self-shaping. Multiple shaped bilayer plates can be stack-laminated and additionally laminated with a thin cold bent locking layer (with same thickness as a passive layer) to form shape-stable curved CLT. (F) Application example as barrel vaulted CLT roof structure with wood fiber direction of thick lamellas (active layers) in load-bearing direction. (G) Application example as curved CLT wall with wood fiber direction of thick lamellas (active layers) in vertical direction.

Using wood, we demonstrate the unique ability of upscaling the self-shaping mechanism of bilayers on the meter scale to obtain large-scale components with high curvature. The shaping process by drying of large wood bilayers is shown in Fig. 1 (A to D), and an exemplary time-lapse video is shown in movie S1. After drying, multiple curved wood bilayer plates can be laminated together to produce curved cross-laminated timber (CLT) plates, which are fully form stable, independent of further changes in moisture (Fig. 1E). Application concepts as efficient CLT roof or wall structural elements are shown in Fig. 1 (F and G). Using this innovative manufacturing approach, material waste by subtractive milling to shape is eliminated, while extensive cold bending of thick lamellae is rendered unnecessary. In addition, higher curvature than in standard form-giving processes is enabled, while, at the same time, thicker wood lamellae can be used. This concept is arbitrarily scalable in any in-plane direction of the CLT plate with respect to the size of available material. We suggest a four-dimensional (4D) production approach (*24*–*26*), where a simulated target element shape enables design of the initial flat-shaped structure in function of boundary conditions (BCs) such as change in wood moisture content (WMC), lamella thickness, or growth ring inclination. The shown approach has the potential to revolutionize mass timber production and application, for which the promotion is seen as a key step toward improving sustainability in the modern building sector (*27*).

To make use of this novel approach, a fundamental understanding of the mechanics of shape change on a large scale is required and will be in focus of this study. Inspired by the analysis of bimetal thermostats (*28*), elastic models for predicting bilayer shape change were developed and adapted for diverse swelling systems (*29*–*33*). In the case of bilayers made out of hardwood species, such models have shown to perform reasonably well in predicting shape change of thin layers of thickness below 10 mm (*14*, *34*). However, they do not give suitable insight into the mechanical behavior of wood bilayers. Too many simplifying assumptions, such as restriction to linear elastic deformation, 2D geometry, or steady-state moisture conditions, are not valid for bulk wood. Under time-dependent loading conditions as well as under simultaneous changes in WMC, wood displays more complex behavior. Under the high residual stress state induced by self-shaping (*34*), phenomenological deformation mechanisms such as viscoelasticity, mechanosorption, and plasticity may simultaneously occur (*35*–*37*). In addition to time- and moisture-dependent mechanical behavior, effects of moisture diffusion due to bilayer drying (or wetting) of exposed surfaces need to be considered. In the bulk, diffusion time is proportional to squared diffusion path when assuming Fickian transport laws. Thus, bilayer equilibration time in the target climate is drastically increased with lamella thickness, and moisture gradients may heavily affect the time-dependent mechanical response.

We address the mentioned issues by 3D finite element (FE) analysis using an elaborate rheological model for wood. Experimental data of self-shaping by drying of three wood bilayer configurations made out of the abundant hard- and softwood species European beech and Norway spruce are shown. Hereby, the total bilayer thicknesses range from 15 to 45 mm. The numerically and experimentally investigated layer thicknesses are chosen in the optimal scale range for industrial timber production. Thus, direct application is enabled without the need of further upscaling studies. To capture influence of the inherent natural variability in material parameters on resulting shape, a global-type sensitivity analysis (*38*) is conducted.

## RESULTS

### Shape change after drying

**Fig. 2:**
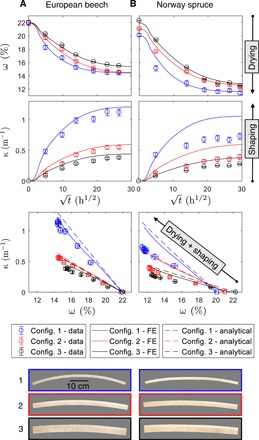
Shape change after drying. Bilayer samples (configurations 1 to 3, depicted at the bottom) made out of beech (**A**) and spruce (**B**) wood during 900-hour drying time. Drying dynamics (WMC, denoted ω) as simulated by the FE models and measured on experimental samples (error bars denote ±SDs). Curvatures (denoted κ) versus square root of time, and curvatures versus moisture contents with comparison to model predictions.

Climate-induced shaping of three investigated bilayer configurations, i.e., increase in curvature and decrease in WMC over time are represented in Fig. 2 for two wood species, European beech and Norway spruce, respectively. The investigated experimental samples showed neither cracking in the bulk wood nor delamination at the bond lines during the shaping process, in which they were relocated from a high to a low relative air humidity (RH) climate at 20°C. WMCs were approximately equilibrated after 400 hours in the dry target climate, except for the bilayer series with a thickness of 45 mm, for which an equilibration is not visible after 900 hours. Beech bilayers reached an equilibrium WMC of around 14.5%, representing a difference of 7.5% as compared to the initial state. Spruce bilayers reached around 12.5% with a difference of approximately 9% in WMC. Despite the larger difference, curvature of spruce bilayers was lower than that of beech bilayers for all three configurations. In the case of beech bilayers (Fig. 2A), curvature is simulated with reasonable accuracy, including light overestimation. In contrast, for spruce (Fig. 2B), considerable overestimation can be recognized. Comparing both beech and spruce FE simulations with the performance of a simple analytical model derived for shape prediction, a matching trend can be observed. The predictions by both models are close and, especially for spruce, do not appear to considerably differ in comparison to the data.

### Stress and strain states

**Fig. 3:**
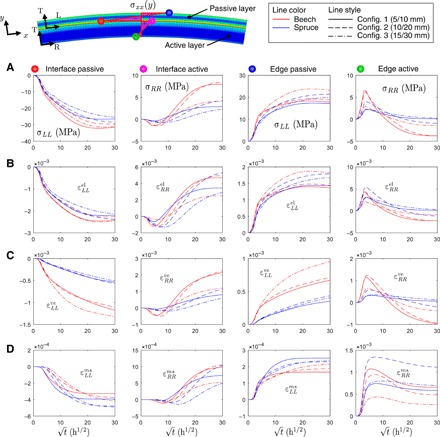
Shaping-induced stresses and strains. (**A**) Bilayer axial stress states (**σ***_xx_*, indices *LL* for passive and *RR* for active layer corresponding to local wood anatomical coordinate system) at four points of interest versus time (*t*) for configurations 1 to 3 and both wood species. Strain state (**ε***_xx_*) separated in individual contributors. (**B**) Elastic ($\varepsilon_{\mathrm{xx}}^{\text{el}}$). (**C**) Viscoelastic ($\varepsilon_{\mathrm{xx}}^{\text{ve}}$). (**D**) Mechanosorption ($\varepsilon_{\mathrm{xx}}^{\text{ms}}$). Irreversible plastic strains ($\varepsilon_{\mathrm{xx}}^{\text{pl}}$) equal zero for all configurations. Total strains (**ε**^tot^) and hygro-expansion strains (**ε**^ω^) are not shown.

Bilayer axial stresses and strains as a function of square root of drying time are shown in Fig. 3 for all configurations and at four relevant points of interest, namely, at the outer edge and interface of both passive and active layer, respectively. Maximum compressive stresses of around 30 MPa for beech and 25 MPa for spruce are found at the passive layer interface. At the passive layer edge, the tensile stresses range between 17 and 24 MPa. No distinct difference between species or any dependency on bilayer thickness of the stresses can be recognized. However, in the active layer, stress is generally found to be higher for beech than for spruce. At the edge of the active layer, tensile stresses up to 6 MPa are developed until approximately 100 hours of drying. After that, they reverse into compression. The opposite pattern is observed at the active layer interface, where final tensile stresses range between 2 and 8 MPa. Across the entire bilayer cross-section and after 900 hours, the stress states show the typical bending stress shape of two bonded layers (*28*).

For the elastic strains, a similar pattern as in the case of the stresses can be observed. The highest elastic strains reach 0.25% in the passive layer and 0.55% in the active layer. No systematic influence of bilayer thickness or species can be seen. The viscoelastic strains are in the same order of magnitude as the elastic strains. However, here, an apparent difference between the two species beech and spruce can be observed. Viscoelastic strains are approximately twice as high for beech than for spruce. Noticeably, the viscoelastic strains do not converge even after 900 hours, while curvature and WMC remain constant after 400 hours already. Mechanosorptive strains for the passive layer are lower by approximately one order of magnitude compared to the other two strain components and show convergence after 900 hours. Plastic strains do not appear for any configuration.

### Sensitivity analysis

A global-type, nonlinear sensitivity analysis for quantification of uncertainty in model input parameters is presented in sections S1 to S3. For beech wood bilayers, variability in simulated shape is obtained as being entirely dependent on variability in the axial swelling (or shrinkage) coefficient of the active layer. In the case of spruce, the variability in axial stiffness of layers appears to dictate variability in shape change.

## DISCUSSION

Self-shaping by drying within the hygroscopic range of wood has been demonstrated for lamellae thicknesses up to 30 mm and for two abundant European wood species, one hardwood and one softwood. Commonly, wooden cross-ply laminates such as CLT experience extensive cracking and delamination due to high environmentally induced residual stresses (*39*). Especially for hardwood species, standard-conform gluing for structural applications is still challenging. To avoid moisture-induced delamination, the application of priming solutions before applying polyurethane adhesive is recommended (*40*, *41*). For the case of self-shaping wood, this is not necessary. The mechanical compatibility of bilayer structures, allowing large unconstrained deformations, prevents delamination even for very high changes in WMC.

The computational model fairly predicted the curvature of beech bilayers, while the spruce bilayer curvature was overestimated for all configurations. Unknown material nonlinearity in wood or inaccuracy in used material parameters may contribute to the observed deviation. In addition, a more accurate model may be provided by considering the next smaller representative elementary volume. At the intra-growthring scale, spruce shows considerably larger inhomogeneity compared to beech because of more pronounced differences in material properties of early- and latewood. Such localized mechanical behavior may considerably affect mechanics at large scale. However, the required material parameters are still missing, and collecting them is a nontrivial task. Considering that Norway spruce is one of the most widely used construction woods, a future clarification is certainly of great interest.

The sensitivity analysis revealed that in the case of beech wood, all model variability can be attributed to the natural variability in swelling coefficients. Therefore, a statistical analysis thereof needs to be conducted to increase model prediction (an example is shown in section S3). The other input parameters are of negligible relevance in terms of reducing uncertainty in shape prediction, given the model is valid as in the case of beech. This finding is relevant for application cases where there is a large apparent variability in quality of available material. In the case of spruce wood, a single dominating parameter could not be identified. Effectively, even by attributing a coefficient of variation of 10% to the most important considered parameters, identified as being the axial elastic layer stiffnesses, total model variation is found to be 4% only (section S1). Because the probability that the model-propagated uncertainty decreases is low, further parameters remain to be investigated to explain the more complex behavior of spruce wood.

A further insight provided by the sensitivity analysis is that the parameters of the adhesive layer, namely, its thickness, stiffness, and shear modulus, do not influence shape change. This can be explained by the fact that in bilayer laminates, the edge stresses dictate deformation due to the bending regime, although stresses are found highest at the interface. The exclusive task of the adhesive bond is to block relative deformation of the two layers with respect to each other. This implies that modeling and accounting for an adhesive layer is not necessary for self-shaping bilayer composites where bond thickness is small compared to the lamina thickness. However, modeling an adhesive bond accounts for correct moisture and drying behavior, as it represents a diffusion barrier.

In terms of predicting shape change alone, simple analytical models proved to be equally suitable even for thick lamellae. However, axial stresses developing over drying time were found considerably lower in the FE analyses than if the analyses were conducted using a 2D linear elastic-only model (*34*). In the FE analyses, relaxation occurred in both passive and active layers simultaneously, seemingly canceling out influence on curvature. Independent of the thickness or species, viscoelastic strains did not fully converge after 900 hours of drying, although curvature change has already ceased before, indicating a layer-wise compensation. As a consequence, mechanical energy dissipation can here not be correlated with shape change. Accordingly, bilayer shape change can be interpreted as originating from a ratio of axial stresses of active to passive layer, and thus is unaffected by stress magnitudes. This can be used to explain the observed accuracy of analytical models that neglect complex deformation mechanisms. In such models, elastic material parameters enter a simple expression in terms of ratios (*14*, *28*).

The FE analyses showed that bilayer thickness does not affect axial stress magnitudes, which confirms findings in (*34*). Thicker wood bilayers resulted in lower curvature solely due to the cross section’s higher second moment of area increasing the bending stiffness. For industrial large-scale application, this implies that any arbitrary lamella thickness can be used in consideration with the trade-off in target curvature. Design principles valid for thin layers at small scale can therefore also be applied at large scale.

It was seen that no plastic strains develop and that the maximum axial stresses for beech and spruce are far lower than theoretical yield stresses or strengths. For beech and spruce, axial stresses in passive layer after 900 hours roughly reach 60% of the theoretical yield stress in L-direction [e.g., at ω = 14.5%, *f*_*c*, *L*_ = 53 MPa for beech (*35*)]. In the active layer, the maximal stresses scale as 70 and 50% of the strengths in R direction for beech and spruce. The fact that no irreversible strains were accumulated can be attributed to the slow drying dynamic allowing time for relaxation. These findings imply that in the case of application of bilayers as climate-regulated actuator elements, shape-change dynamics and shape reversibility are affected by rate-dependent deformation mechanisms. This may be relevant for any dynamic bilayer structure made from biological materials. However, as mentioned above, the final shape in a state where moisture- and time-dependent deformation mechanisms converged is mainly influenced by factors affecting stress state and bending stiffness, such as layer thicknesses, expansion coefficients, or elastic material properties.

Moisture gradients were shown to have substantial effects on the developing stresses and strains. This was demonstrated in Fig. 3 where, for the case of the active layer edge, stress and strain states inverted over the drying time. The outer active layer edge, which is drying first, will shrink and develop tensile stresses and set the still wet core under compression. Later in time, when moisture gradients decrease, bending regime takes over as the bilayer bends and stresses invert to compression. A critical moment is identified when the strongest moisture gradients, caused by BC-driven drying, are created. For the investigated samples, this critical time can be identified as being 10 to 20 hours after climate relocation. There, the gradient-induced tensile stresses of the active layer edges are maximal and close to theoretical strength values. A fast drying procedure creating sharp moisture gradients may thus lead to cracking in the active layer. When using drying kilns at industrial scale, a mild drying procedure is thus recommended to avoid potential cracks, which would affect target curvature.

### Summary

Self-shaping was presented as a novel concept for industrial production of form-stable curved mass timber elements. The combination of a computational mechanical analysis, a sensitivity analysis, and an experimental investigation has revealed insights into the complex behavior of the self-shaping mechanism of wood. Specific large-scale problems, such as predictability of shape, sensitivity to natural variation in material properties, or drying procedure were addressed and discussed for two wood species, for which considerable differences were identified. Target curvature remains constant if layer stress ratios remain balanced under the influence of different deformation mechanisms. Creep mechanisms prevent exceeding strengths and yield stresses during shaping. Axial stress levels remain independent of bilayer thickness given that the layer thickness ratio is the same. A critical moment is reached when axial stresses in the active layer are of tensile nature due to the initial BC-dictated drying phase. The findings enable application of the biomimetic self-shaping of wood at large scale and promote its integration into mass timber industry.

## MATERIALS AND METHODS

### Constitutive material model for wood

**Fig. 4:**
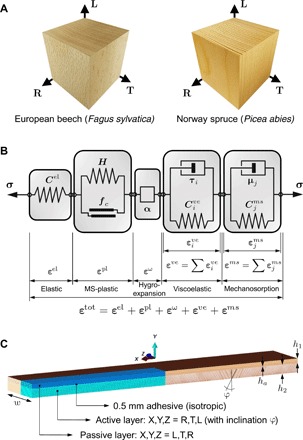
Simulation model. (**A**) Representative elementary volume for wood material model (beech and spruce wood) with anatomical growth directions R, T, and L. (**B**) Schematic rheological model for bulk wood as in (*35*). (**C**) Setup and BC for the FE bilayer models (quarter model with XY and YZ symmetry). *C*, stiffness matrices of springs for respective deformation modes; *H*, hardening moduli; *f_c_*, yield functions; α, differential swelling coefficients; τ*_i_*, characteristic retardation times; μ*_j_*, moisture analogous to τ*_i_*; **σ** and **ε**, stress and strain tensors.

Bulk wood was modeled using a 3D orthotropic, moisture- and time-dependent constitutive material model. The model is based on a representative elementary volume as shown in Fig. 4A. It considers all up-to-date known deformation mechanisms in coupled manner by additive decomposition of the total strain tensor assuming infinitesimal strain theory. Following (*42*), the Helmholtz free energy function Ψ is defined as

$$\begin{array}{c} \Psi =\frac{1}{2}\varepsilon^{\text{el}}C^{\text{el}}\varepsilon^{\text{el}}+\frac{1}{2}\sum_{i=1}^{n}\varepsilon_{i}^{\text{ve}}C_{i}^{\text{ve}}\varepsilon_{i}^{\text{ve}}\\ +\frac{1}{2}\sum_{j=1}^{m}\varepsilon_{j}^{\text{ms}}C_{j}^{\text{ms}}\varepsilon_{j}^{\text{ms}}+\frac{1}{2}\sum_{l=1}^{r}q_{l}\alpha_{l} \end{array}$$ (1)

The Cauchy stress tensor **σ** acting on a material point is in relation to the total strain tensor **ε**^tot^ by **σ** = ∂Ψ/∂**ε**^tot^, where **ε**^tot^ decomposes as shown in Fig. 4B into elastic, plastic, viscoelastic, mechanosorptive, and swelling and shrinkage strain components. In the first term of Eq. 1, **C**^el^ represents the elastic stiffness tensor. $C_{i}^{\text{ve}}$ is the *i*th viscoelastic stiffness tensor, and $C_{j}^{\text{ms}}$ is the *j*th mechanosorptive tensor of the respective Kelvin-Voigt rheological elements connected in series (*n* = 4 and *m* = 3) (Fig. 4B). The last term in Ψ considers the isotropic hardening energy standing for irrecoverable plastic deformation accumulated by a multisurface plasticity model (*r* denotes the number of active yield mechanisms, *q_l_* denotes the plastic hardening functions, and α*_l_* denotes the internal hardening variables for *l* = R,T,L). All element entries of the mentioned compliance tensors (**C**^−1^) for the species European beech and Norway spruce are considered as functions of moisture ω. Further, hygro-expansive (**ε**^ω^) and elastic (**ε**^el^) deformations are rate independent, and **ε**^ω^ is independent on **σ**. A detailed description of the material model, used material parameters, and numerical implementation into the FE framework Abaqus CAE as a user material subroutine is found in (*35*, *36*).

### Computational model

Wood bilayers were modeled using the FE method with the above described material model. The geometrical model and BCs are presented in Fig. 4C. The dimensions were chosen according to the experimental sample set described below. The interface region, acting as a diffusion barrier, was modeled by a 0.5-mm-thick and isotropic one-component polyurethane adhesive (1cPUR) by tie connection. The moisture diffusion process (BC for surface moisture flux) was inversely fitted to experimental data of average moisture content evolution over time by comparing the volume-weighted average WMC. Analogy between temperature and moisture was made for modeling transient diffusion using Fick’s second law; ∂ω/*∂t* = ∇ (**D** ∇ ω) is equivalent to (ρ*c_T_*)*∂T*/*∂t* = ∇ (**K** ∇ *T*) when ρ*c_T_* = 1 so that the matrix of diffusion coefficients *D* equals the matrix of thermal conductivity coefficients *K*. In a first step, heat transfer analyses were conducted using 20-node quadratic brick elements. The resulting temperature evolution fields were then used as predefined fields for static analyses with same mesh and elements. A large deformation theory was applied. The resulting bilayer curvature was calculated as $\kappa =-2u_{y}{\left(u_{y}^{2}+(l+u_{x}^{2})\right)}^{-1}$, using the tip displacements *u_x_* and *u_y_* and assuming a uniform circle-arc-segment–shaped bilayer of initial length *l*.

### Analytical model

An analytical model, derived in (*34*), was used to alternatively model the investigated wood bilayer configurations. The model follows Timoshenko’s work on bimetal thermostats (*28*) but further represents the anisotropic and moisture-dependent material behavior of wood in 2D by assuming a plane-strain state. A linear elastic deformation mechanism is considered exclusively.

### Experimental samples

Using European beech (*Fagus sylvatica*) and Norway spruce (*Picea abies*) wood conditioned at 95 and 85% RH, respectively, three bilayer configurations were produced for each species. Beech was conditioned in adsorption and spruce in desorption equilibrium from the green state. This setup targeted a similar initial moisture content for comparability of both species. The used wood was defect free and cut from the same stem. A scheme of the bilayer setup is given in Fig. 4C, where the local wood anatomical orientations R,T,L are given in terms of global orientations X,Y,Z for passive (layer 1, top) and active (layer 2, bottom) layers. The components were bonded using 1cPUR adhesive (HB S309 Purbond, Henkel & Cie. AG, Switzerland), as curing by poly-addition enables gluing at high moisture contents. A constant thickness ratio of *h*_1_ : *h*_2_ = 1 : 2 (passive:active) was maintained in all configurations. The total thicknesses *h*_1_ + *h*_2_ of the three configurations were chosen to be 15 mm (configuration 1), 30 mm (configuration 2), and 45 mm (configuration 3). Width and length of bilayers were chosen to be 100 and 600 mm. In the active layers, growth ring inclinations (φ) of 0° to 20° for the beech and 0° to 30° for the spruce were measured on reference samples (details in section S3). After production in initial climate, the beech samples were relocated into 65% RH and the spruce samples into 50% RH climate for drying. Curvature and weight were measured over 900 hours of acclimatization time on eight samples per configuration. Curvature was calculated as κ = ψ′′(1 + ψ′^2^)^−3/2^ by image analysis. Second-order polynomials ψ were fitted to thresholded edge segments obtained by applying a Canny edge detector algorithm. WMCs ω were determined using reference samples cut from the bilayer samples before climate relocation (details in section S3).

## Supplementary Material

http://advances.sciencemag.org/cgi/content/full/5/9/eaax1311/DC1

Download PDF

Movie S1

Analysis of hygroscopic self-shaping wood at large scale for curved mas timber structures

## SUPPLEMENTARY MATERIALS

Supplementary material for this article is available at http://advances.sciencemag.org/cgi/content/full/5/9/eaax1311/DC1 Movie S1. Time-lapse video of large-scale wood bilayer actuation. Section S1. Sensitivity analysis Section S2. The sensitivity parameter $S_{i}^{\text{tot}}$ Section S3. Statistical analysis of shrinkage coefficient in active layer Fig. S1. Results of sensitivity analyses. Fig. S2. Statistical analysis of shrinkage coefficient. Table S1. Input and output of uncertainty quantification. Table S2. Statistical test results on differential swelling coefficient measurements. References (*43*–*47*)
