# Supplementary material for: Analysis of hygroscopic self-shaping wood at large scale for curved mass timber structures
Source: Sci Adv. 2019 Sep 13;5(9):eaax1311. doi: 10.1126/sciadv.aax1311 (PMC6744262; doi:10.1126/sciadv.aax1311)
Supplement: http://advances.sciencemag.org/cgi/content/full/5/9/eaax1311/DC1 [file supp_5_9_eaax1311__index.html]

Science Advances | Science AdvancesAAASSearchScience AdvancesMenu

## Supplementary Materials

**The PDF file includes:**

- Legend for movie S1
- Section S1. Sensitivity analysis
- Section S2. The sensitivity parameter Sitot
- Section S3. Statistical analysis of shrinkage coefficient in active layer
- Fig. S1. Results of sensitivity analyses.
- Fig. S2. Statistical analysis of shrinkage coefficient.
- Table S1. Input and output of uncertainty quantification.
- Table S2. Statistical test results on differential swelling coefficient measurements.
- References (*43*–*47*)

Download PDF

**Other Supplementary Material for this manuscript includes the following:**

- Movie S1 (.mp4 format). Time-lapse video of large-scale wood bilayer actuation.

**Files in this Data Supplement:**

- Adobe PDF - aax1311\_SM.pdf
