# Supplementary material for: Analysis of hygroscopic self-shaping wood at large scale for curved mass timber structures
Source: Sci Adv. 2019 Sep 13;5(9):eaax1311. doi: 10.1126/sciadv.aax1311 (PMC6744262; doi:10.1126/sciadv.aax1311)
Supplement: Download PDF [file aax1311_SM.pdf]

Science Advances
AAAS

advances.sciencemag.org/cgi/content/full/5/9/eaax1311/DC1

Supplementary Materials for

Analysis of hygroscopic self-shaping wood at large scale for curved mass timber structures

Philippe Grönquist*, Dylan Wood, Mohammad M. Hassani, Falk K. Wittel, Achim Menges, Markus Rüggeberg*

*Corresponding author. Email: philippe.groenquist@empa.ch (P.G.); markus.rueggeberg@empa.ch (M.R.)

Published 13 September 2019, *Sci. Adv.* **5**, eaax1311 (2019)
DOI: 10.1126/sciadv.aax1311

The PDF file includes:

Legend for movie S1
Section S1. Sensitivity analysis
Section S2. The sensitivity parameter S_i^{tot}
Section S3. Statistical analysis of shrinkage coefficient in active layer
Fig. S1. Results of sensitivity analyses.
Fig. S2. Statistical analysis of shrinkage coefficient.
Table S1. Input and output of uncertainty quantification.
Table S2. Statistical test results on differential swelling coefficient measurements.
References (*43–47*)

Other Supplementary Material for this manuscript includes the following:

(available at advances.sciencemag.org/cgi/content/full/5/9/eaax1311/DC1)

Movie S1 (.mp4 format). Time-lapse video of large-scale wood bilayer actuation.

Movie S1. Time-lapse video of large-scale wood bilayer actuation.

Video Caption

Shape-change of a large-scale Norway spruce bilayer plate with total thickness of 31.5 mm (passive and active layer thicknesses of 6.5 and 25 mm), length of 1.5 m, and width of 0.5 m. Production in initial flat shape with wood conditioned at 85% RH. Self-shaping in 35% RH and room temperature over 270 hours.

Section S1. Sensitivity analysis

Methods

A sensitivity analysis using total Sobol' indices S_i^{tot} was conducted on the FE models of the wood bilayers. S_i^{tot} reveal how much an attributed uncertainty, i.e. a distribution function of a certain model input parameter contributes to the final model output distribution. The computational costs of the FE models did not allow for a standard Monte-Carlo (MC) sampling approach in order to conduct the sensitivity analyses. Using a MC based calculation, the convergence rate is $\propto N^{-1/2}$, where N is the number of MC samples, i.e., required model evaluations. A convenient surrogating approach is to replace the model by a set of sparse truncated polynomial chaos (PC) expansions [43]. The derivation of the sensitivity measure S_i^{tot} from PC expansion is described in supplementary S3. Here, bilayer curvature after 900 hours represents the model response ($Y = \kappa$) for beech and spruce bilayers drying from 21% to 14% WMC. The selected input variables (their number M is limited, even for sparse PC approaches) with associated uncertainty were divided in three categories per wood species such that six sensitivity analyses were conducted, denoted UQ 1-6. Odd and even numbering correspond to beech and spruce bilayers respectively.

- UQ 1 & 2: The influence of the variability in geometry is analyzed together with variability in adhesive material properties. Uncertainty was attributed to the bilayer thicknesses (h_1 and h_2) and width (w), the adhesive layer thickness (h_a), the growth ring orientation in active layer (φ), and to the Young's and shear moduli of adhesive (E_a and G_a, for 1cPUR at $\omega = 0$).
- UQ 3 & 4: Here, variability was attributed only to the independent engineering constants in the wood elastic compliance tensor $\boldsymbol{C}^{el}$, i.e. to E_R, E_T, E_L, G_{RT}, G_{RL}, G_{TL}, ν_{TR}, ν_{LR}, ν_{LT}, at $\omega = 0$.
- UQ 5 & 6: Finally, the most relevant parameters from UQ 1-4 were chosen along with the three differential swelling coefficients α_R, α_T, and α_L.

All input parameters were assumed as being independent random variables following a specific probability density function (PDF) with parameters as given in Tab. S1. Geometrical parameters were assumed to follow a Gaussian PDF ($\mathcal{N}$) and material properties a lognormal PDF ($\mathcal{LN}$). An updated probabilistic input model, namely a three-parameter Burr Type XII PDF ($\mathcal{B}$XII) was chosen for α_R. The parameters of the updated PDF were experimentally determined and the statistical analysis is described in supplementary S4 (Fig. S2 and Tab. S2).
The PC expansion was conducted using the *UQlab* framework in *Matlab* [44]. The MC-sampled set of input parameters is transferred from *Matlab* to parametric *Python* (FE model) and *Fortran 77* (material model) scripts for the automated UQ and FE analysis via parameter interfacing scripts. The obtained simulated data set, chosen as 150 FE model evaluations for each sensitivity analysis, allowed calculation of the sparse PC expansions with a total polynomial degree of $p = 5$ using a least angle regression method.

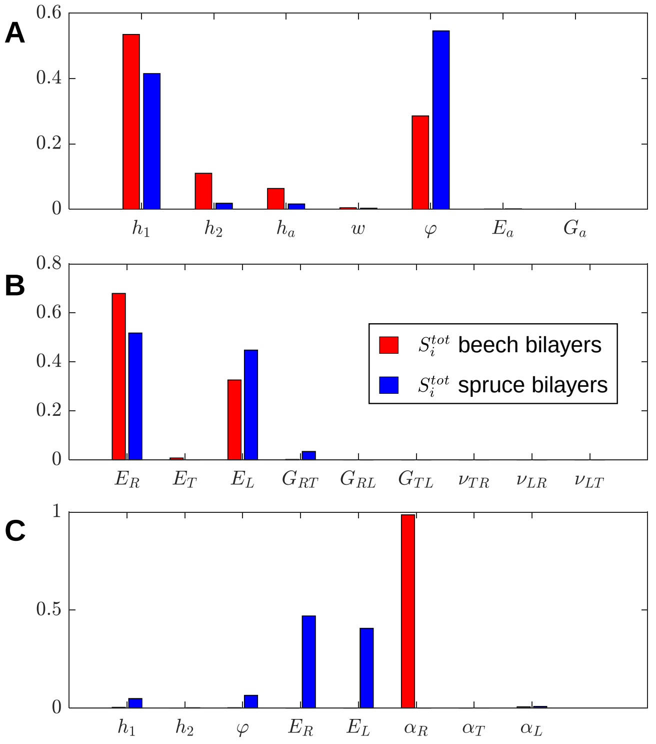

Fig. S1. Results of sensitivity analyses. Values of total Sobol' indices S_i^{tot} for UQ 1-6 for Config. 2 of beech (red) and spruce (blue) bilayers. UQ 1 and 2 (A), UQ 3 and 4 (B), and UQ 5 and 6 (C).

Results

The total Sobol' indices S_i^{tot} from the six conducted UQs are displayed in Fig. S1 and the corresponding model output Y can be found in Tab. S1. From UQ1 and UQ2, it can be seen that variations in adhesive properties (E_a and G_a) completely vanish in terms of output κ and that in the same analyses h_1 and φ are mostly responsible for variations in κ. For beech, h_2 and h_a are attributed minor importance as well. In the second analyses (UQ3 and UQ4) only the axial layer stiffness E_R (active layer) and E_L (passive layer) seem to be of relevance. These values, along with the swelling coefficients were chosen for the third set of analyses, UQ5 and UQ6. Here, considerable differences are visible between the two species, as for beech, only variation in α_R seems to impact variation in κ. On the other hand, for spruce, the layer stiffnesses appear to be the most important model input parameters.

Table S1. Input and output of uncertainty quantification. Model input parameters (X_i) with used sampling parameters (i.e., attributed uncertainty μ and COV, and with PDFs $\mathcal{N}$, $\mathcal{LN}$, and $\mathcal{B}XII$) and model output (Y) for the six conducted sensitivity analyses. First moments (μ) represent ordinate axis intercept of moisture dependent function of the wood independent engineering constants taken from [35], i.e., at $\omega = 0$. UQ 1,3, and 5 for beech bilayers (black font), and UQ 2,4, and 6 for spruce bilayers (gray font).

	X_i									Y
UQ 1 & 2	h_1	h_2	h_a	w	φ	E_a	G_a			κ
μ	10 mm	20 mm	0.5 mm	100 mm	10°	754 MPa	290 MPa			0.55 mm^{-1}
	10 mm	20 mm	0.5 mm	100 mm	10°	754 MPa	290 MPa			0.42 mm^{-1}
COV	1%	0.5%	10%	0.5%	10%	10%	10%			0.73%
	1%	0.5%	10%	0.5%	10%	10%	10%			1.38%
PDF	$\mathcal{N}$	$\mathcal{N}$	$\mathcal{N}$	$\mathcal{N}$	$\mathcal{N}$	$\mathcal{LN}$	$\mathcal{LN}$			
UQ 3 & 4	E_R	E_T	E_L	G_{RT}	G_{RL}	G_{TL}	ν_{TR}	ν_{LR}	ν_{LT}	κ
μ	2566 MPa	885 MPa	17137 MPa	668 MPa	1482 MPa	1100 MPa	0.29	0.38	0.34	0.55 mm^{-1}
	1000 MPa	506 MPa	12792 MPa	61 MPa	763 MPa	881 MPa	0.15	0.23	0.29	0.42 mm^{-1}
COV	10%	10%	10%	10%	10%	10%	10%	10%	10%	1.77%
	10%	10%	10%	10%	10%	10%	10%	10%	10%	3.79%
PDF	$\mathcal{LN}$	$\mathcal{LN}$	$\mathcal{LN}$	$\mathcal{LN}$	$\mathcal{LN}$	$\mathcal{LN}$	$\mathcal{LN}$	$\mathcal{LN}$	$\mathcal{LN}$	
UQ 5 & 6	h_1	h_2	φ	E_R	E_L	α_R [a]	α_T	α_L		κ
μ	10 mm	20 mm	10°	2566 MPa	17137 MPa	$0.00178\%^{-1}$	$0.0040\%^{-1}$	$0.00011\%^{-1}$		0.55 mm^{-1}
	10 mm	20 mm	10°	1000 MPa	12792 MPa	$0.00169\%^{-1}$	$0.0033\%^{-1}$	$0.00005\%^{-1}$		0.42 mm^{-1}
COV	1%	0.5%	10%	10%	10%	7.56%	10%	10%		8.33%
	1%	0.5%	10%	10%	10%	13.86%	10%	10%		3.99%
PDF	$\mathcal{N}$	$\mathcal{N}$	$\mathcal{N}$	$\mathcal{LN}$	$\mathcal{LN}$	$\mathcal{B}$XII	$\mathcal{LN}$	$\mathcal{LN}$		

[a] Values as determined in Fig. S2 and Tab. S2

Section S2. The sensitivity parameter S_i^{tot}

Any computational model $\mathcal{M}$ with model-response random vector $Y = \mathcal{M}(\boldsymbol{X})$ and input random vector $\boldsymbol{X} \sim f_{\boldsymbol{X}}$, where $f_{\boldsymbol{X}}$ is the product of each marginal distribution assuming a set of M independent input parameters, can be expressed using a spectral approach [45, 46]. The response Y is considered as point in a function space of square integrable functions ($\mathbf{L}^2$) with coordinates y_α. Hereby, the set of multivariate polynomials $\{\Psi_\alpha, \alpha \in \mathbb{N}^M\}$ form an orthonormal basis in $\mathbf{L}^2$. The (M-dimensional) multivariate basis is given by

$$\Psi_\alpha(\boldsymbol{x}) := \prod_{i=1}^{M} \Psi_{\alpha_i}^{(i)}(x_i) \tag{S1}$$

and the model response can be written as

$$Y = \mathcal{M}(\boldsymbol{X}) = \sum_{\alpha \in \mathbb{N}^M}^{\infty} y_\alpha \Psi_\alpha(\boldsymbol{X}) \approx \sum_{\alpha \in \mathcal{A}^{M,p}} y_\alpha \Psi_\alpha(\boldsymbol{X}) \tag{S2}$$

where $\boldsymbol{\alpha} \in \mathbb{N}^M$ is a multi-index of dimension M and $\mathcal{A}^{M,p}$ defines a sparse truncated basis set of total polynomial degree p. The residual resulting from the truncation is minimized by a least-square method. The finite number of coefficients y_α of the polynomial for the truncated expansion can, due to the orthogonality of the basis, be individually calculated by projection of $\mathcal{M}$ onto Ψ_α

$$y_\alpha =< \mathcal{M}(\boldsymbol{X}), \Psi_\alpha(\boldsymbol{X}) >= \int_{\mathcal{D}_X} \mathcal{M}(\boldsymbol{x})\Psi_\alpha(\boldsymbol{x}) f_{\boldsymbol{X}}(\boldsymbol{x}) d\boldsymbol{x} \tag{S3}$$

where $\mathcal{D}_{\boldsymbol{X}}$ is the support of the probability density function $f_{\boldsymbol{X}}$. The integral can be evaluated by quadrature where the chosen type of polynomial is orthogonal with respect to the corresponding weight function w such that $w \equiv f_{\boldsymbol{X}}$. A limited number of model evaluations is sufficient for calculating y_α. The obtained PC expansion can then be rearranged to read analogous to a Sobol' decomposition [47]. Total Sobol' indices S_i^{tot} for input variable X_i of the model can, thus, be directly computed with the obtained coefficients y_α as

$$S_i^{tot} = D_i^{tot}/D = \sum_{\alpha \in \mathcal{A}_i^{tot}} y_\alpha^2/D \tag{S4}$$

such that $\mathcal{A}_i^{tot} = \{\boldsymbol{\alpha} \in \mathbb{N}^M : \alpha_i > 0\}$, D_i^{tot} is the partial variance (sum over all orders) of parameter X_i, and D is the variance of Y ($D = Var[\mathcal{M}(\boldsymbol{X})]$).

Section S3. Statistical analysis of shrinkage coefficient in active layer

Measurement and determination of coefficient α^{φ}

Differential shrinkage coefficients (also referred to as swelling, moisture expansion or contraction coefficients) were measured on 207 reference samples of beech and 132 of spruce. The samples were cut from the active layers of the investigated bilayer samples as cuboid samples with edge length corresponding to thickness of active layers (for config. 1-3, 10, 20, and 30 mm) and length of 50 mm in R-direction (or inclined R-direction, by an angle φ). The samples were cut at 95% RH for beech and at 85% RH for spruce and relocated for 4 weeks to 65% RH and 50% RH respectively. The samples were weighted and sample dimensions were measured in initial state, after reaching desorption equilibrium after 4 weeks in dry climate, and after oven-drying the samples for 48 hours at 103°C. Differential shrinkage coefficients were calculated as $\alpha = \varepsilon^{\omega}\Delta\omega^{-1}$ where ε^{ω} is the recorded shrinkage strain. The moisture contents ω were calculated as $\Delta\omega = (m_{\omega} - m_0)m_0^{-1}$ where m_{ω} and m_0 are the sample masses at moisture level ω and at oven-dry state.

Derivation of Probabilistic Model for α_R

The measured differential shrinkage coefficients (α^{φ}) are shown in Fig. S2 as a function of growth ring inclination φ. A linear and a strain tensor rotation model were fitted to the data and compared in order to correct for the inclination φ and to transform α^{φ} to coefficients in principal anatomical R-direction α_R, where $\varphi = 0$. The linear transformation, $\alpha^{\varphi} - 2(\alpha_T - \alpha_R)\pi^{-1}\varphi$, was chosen. A slightly skewed distribution of data points (Fig. S2C) resulted from the measurements of shrinkage coefficients. A two-parameter log-normal ($\mathcal{LN}$) and a three-parameter Burr type XII ($\mathcal{B}$XII) distribution were fitted to the transformed data α_R. A better fit was obtained using the Burr distribution as demonstrated by a Likelihood-ratio test shown in Tab. S2, which ruled out the effects of the additional fitting parameter. A value of $\mu_{\alpha_R} = 0.00178$ %$^{-1}$ with COV=7.56% was obtained for beech shrinkage and $\mu_{\alpha_R} = 0.00169$ %$^{-1}$ with COV=13.86% for spruce shrinkage, both characterized by the Burr type XII distribution with parameters as shown in Tab. S2. Results show a high variability in the derived probabilistic model. However, these results are in accordance with natural variability typically found in biological materials, especially in wood.

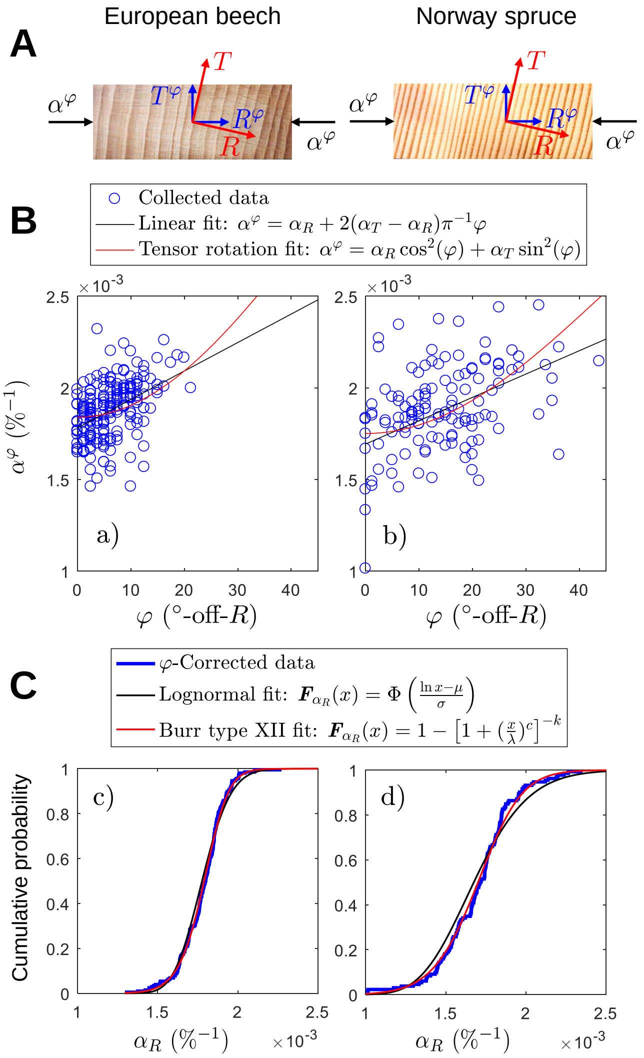

Fig. S2. Statistical analysis of shrinkage coefficient. A: Scheme of material axes and measurement directions. B: Measured differential swelling coefficients α^{φ} vs. growth ring inclination φ for beech and spruce wood reference samples along best fit lines for linear regression (black line) and for strain tensor rotation (red line). C: Cumulative probability plot of corrected differential swelling coefficients α_R by linear regression to $\varphi = 0$ for reference samples along log-normal cumulative distribution fit (black line) and Burr cumulative distribution fit (red line).

Table S2. Statistical test results on differential swelling coefficient measurements. Complementary to fig. S2. Goodness of fit of linear regression and tensor rotation fit to data and parameters of fitted distributions to φ-corrected data. A likelihood-ratio test is used to compare fit of two PDFs having a different number of parameters. Values for beech (black font) and spruce (gray font).

Fit type	Data	Parameter 1	Parameter 2	Parameter 3	GOF[a]	μ_{α_R} ($\%^{-1}$)	σ_{α_R} ($\%^{-1} \times 10^{-4}$)	COV[b] (%)
		α_R ($\%^{-1}$)	α_T ($\%^{-1}$)		SSE[c] ($\%^{-2} \times 10^{-6}$)			
Linear regression	α^{φ}	0.00178	0.00318		3.88			
		0.00169	0.00284		7.86			
Tensor rotation	α^{φ}	0.00184	0.00400[d]		4.03			
		0.00175	0.00330[d]		8.37			
					$\log \mathcal{L}(\theta^{MLE})$[e]			
Log-normal distribution	α_R	$\lambda = -6.33$	$\zeta = 0.0793$		1528	0.00178	1.42	7.94
		$\lambda = -6.392$	$\zeta = 0.159$		899	0.00169	2.72	16.03
Burr Type XII distribution	α_R	$\lambda = 0.00189$	$c = 19.1$	$k = 2.33$	1537	0.00178	1.35	7.56
		$\lambda = 0.00189$	$c = 10.2$	$k = 2.47$	914	0.00169	2.35	13.86
		G^2 [g]	C_α [h]		p-value[i]			
Likelihood-ratio test[f]		18.44	7.815		3.568×10^{-4}			
		29.09	7.815		2.148×10^{-6}			

[a] Goodness of fit; [b] Coefficient of variation; [c] Sum of squared errors
[d] Preassigned values for physical meaning of tensor rotation
[e] Abs. log-likelihood of maximizing parameters θ^{MLE}
[f] Burr dist. fits data better if $G^2 = 2\,[\log \mathcal{L}(\lambda, c, k) - \log \mathcal{L}(\lambda, \zeta)] > C_\alpha$
[g] Statistic of test; [h] Test condition ($\sim \chi^2$) at significance level $\alpha = 0.05$; [i] Test confidence level
